# Benefits and Costs of Happy Entrepreneurs: The Dual Effect of Entrepreneurial Identity on Entrepreneurs' Subjective Well-Being

**DOI:** 10.3389/fpsyg.2021.767164

**Published:** 2021-10-29

**Authors:** Hongtao Yang, Lei Zhang, Yenchun Jim Wu, Hangyu Shi

**Affiliations:** ^1^School of Business Administration, Huaqiao University, Quanzhou, China; ^2^Graduate Institute of Global Business and Strategy, National Taiwan Normal University, Taipei, Taiwan; ^3^Business Management, National Sun Yat-sen University, Kaohsiung, Taiwan

**Keywords:** subjective well-being, entrepreneurial identity, work-related problem-solving pondering, work-related affective rumination, mindfulness

## Abstract

Entrepreneurship research generally focuses more on the entrepreneurial outcomes of entrepreneurs and less on their entrepreneurial process. To a certain extent, well-being reflects how tired entrepreneurs are during entrepreneurship. Based on conservation of resources theory, this study proposes a double-edged sword model of the effect of entrepreneurial identity on subjective well-being, using the two-dimensional structure of work rumination as a mediator. This study also concentrates on the moderating role of entrepreneurial mindfulness. Multiple hierarchical regression methods are used to analyze and test 882 valid samples. Results suggest that the effect of entrepreneurial identity produces distinctly different outcomes. On the one hand, entrepreneurial identity induces entrepreneurs' work-related affective rumination to reduce their subjective well-being through the path of resource depletion. On the other hand, entrepreneurial identity stimulates entrepreneurs' contemplation on work-related problem-solving pondering to enhance their subjective well-being through the path of resource acquisition. In the path of resource depletion, work-related affective rumination produces a “suppressing effect” between an entrepreneur's identity and entrepreneurial subjective well-being. In addition, entrepreneurial mindfulness weakens the resource depletion path. Entrepreneurial mindfulness negatively moderates the relationship between entrepreneurial identity and work-related affective rumination. Entrepreneurial mindfulness also does not strengthen the resource acquisition path. Mindfulness does not positively moderate the relationship between entrepreneurial identity and work-related problem-solving pondering. The findings further extend the research on the influence of entrepreneurial identity on subjective well-being. They also reveal the mechanisms and boundary conditions of the effect of entrepreneurial identity on subjective well-being.

## Introduction

Entrepreneurial well-being is not only a key part of evaluating entrepreneurial achievements but also an important motivation for continuous entrepreneurship after entrepreneurial failure. Entrepreneurial well-being is regarded as a decisive symbol of entrepreneurial success. Entrepreneurs with high well-being can not only better cope with the challenges of work stress and high entrepreneurial failure rate but also show a better level of innovation performance (Stephan, 2018; Yu et al., 2018). For entrepreneurial success, the physical and mental health of entrepreneurs are as important as the development of entrepreneurial enterprises (Uy et al., 2017). However, existing entrepreneurship research focuses more on the economic performance brought about by entrepreneurship and less on whether entrepreneurs are satisfied with their entrepreneurship. Scholars emphasize that entrepreneurship research is more concerned with how high entrepreneurs “fly” and rarely with how tired entrepreneurs “fly” (Yu et al., 2018). Exploring the formation of entrepreneurial well-being can help in understanding not only entrepreneurial results in terms of objective performance but also entrepreneurial success or failure in terms of subjective utility. Previous studies find that compared with employed individuals, entrepreneurs face unstable incomes, high entrepreneurial risks, and great pressure (Uy et al., 2013). Nevertheless, entrepreneurial activities bring well-being to entrepreneurs (Wach et al., 2021). In entrepreneurial practice, some entrepreneurs make a comeback due to failure, whereas others retire at the peak of their careers. What role well-being plays in entrepreneurship needs to be answered by research on entrepreneurial well-being. The subjective well-being of entrepreneurs has attracted attention in academic circles. Scholars define it as entrepreneurs' overall assessment and subjective feelings about their quality of life, job satisfaction, and personal growth and development during the creation and operation of the business. At present, domestic and foreign scholars find that entrepreneurs' subjective well-being can be improved by entrepreneurial characteristics (Hmieleski and Sheppard, 2019) and entrepreneurial context (Abreu et al., 2019; Fritsch et al., 2019; Xu et al., 2021).

A systematic review of the literature reveals that previous research on the antecedent variables of entrepreneurial well-being is conducted in two main aspects. First, scholars explore the impact of “whether entrepreneurial needs are met” on entrepreneurial well-being on the basis of difference theory. Relevant research studies entrepreneurial well-being in chronological order from the satisfaction of entrepreneurial expectations, the dissatisfaction of entrepreneurial expectations (e.g., entrepreneurial failure), and the influencing factors of entrepreneurial expectations (e.g., human capital). Second, based on the job characteristic model, scholars explore the impact of family–work conflict and entrepreneurial heterogeneity on entrepreneurial well-being. Most of the existing studies focus on the “result effect” of entrepreneurship to explore the “positive side” of the antecedent variables on entrepreneurial well-being. The “negative side” of the antecedent variables affecting entrepreneurial well-being is rarely explored using the “process effect” of entrepreneurship. Additionally, there is no systematic discussion on whether the antecedent variables have both positive and negative effects. The entrepreneurial process effect emphasizes that entrepreneurs not only pay attention to entrepreneurial results in the entrepreneurial process but also pay more attention to the conditions for the realization of entrepreneurial results. For example, although entrepreneurs encounter entrepreneurial failures, entrepreneurs may gain personal abilities, social recognition, and social network. This greatly reduces or even offsets the impact of entrepreneurial failure on entrepreneurs' well-being. During entrepreneurship, entrepreneurs usually actively recognize, experience, and evaluate entrepreneurial and internalized entrepreneurial roles to construct and adjust their self-identity of entrepreneurial roles and to be affirmed and recognized by the external environment. This process is named “entrepreneurial identity” (Hekman et al., 2009; Guo et al., 2019). Entrepreneurial identity is the antecedent factor affecting entrepreneurial success and improving entrepreneurial performance. Theoretically, a relationship exists between the identity of entrepreneurs and their subjective well-being. Self-perceptions of entrepreneurial role can enhance entrepreneurial identity aspirations and predict entrepreneurial discovery and exploration behaviors (Farmer et al., 2011). Entrepreneurial identity is committed to entrepreneurial success, and whether or not to start a business can affect an individual's subjective well-being. In summary, studying the impact of entrepreneurial identity on entrepreneurs' subjective well-being has great theoretical and practical significance.

Previous studies mainly investigate the influence mechanism of entrepreneurs' subjective well-being from the perspective of difference theory and self-determination theory. Difference theory suggests that individual satisfaction depends on the difference between actual and predetermined standards. According to this theory, the higher the goals an entrepreneur sets, the harder it is to feel well-being. Some scholars challenge this theory. They find that entrepreneurs with higher expectations of business success have higher satisfaction (Cooper and Artz, 1995). Nonetheless, most research supports the view that “the lower the expectation, the happier they are.” If entrepreneurship fails, does that mean that entrepreneurial expectations have failed? The reality is that some entrepreneurs will be relieved and view it like “euthanasia” after encountering entrepreneurial failure (Yu et al., 2018). Self-determination theory provides a supplementary explanation to difference theory. Self-determination theory suggests that an individual's well-being is derived from the satisfaction of three main needs: autonomy, competence, and belonging. If entrepreneurs' needs for autonomy, competence, and belonging are not met before the failure of the venture, then once the entrepreneurship fails, this will exacerbate the negative emotions such as disappointment and stress caused by the entrepreneurs' psychological fallout and thus affect entrepreneurial well-being (Shepherd and Cardon, 2009). Although corresponding research considers the influence of entrepreneur autonomy on entrepreneurial well-being in the entrepreneurial process, studies focusing on job characteristics still cannot fully demonstrate the important influence of entrepreneurial “process effects” on entrepreneurial well-being. Well-being in the entrepreneurial process emphasizes entrepreneurs' emotional experience and cognitive evaluation. Although difference theory and self-determination theory describe entrepreneurs' well-being based on the “result effect” of entrepreneurship, they cannot explain the subjective well-being of the “process effect” of entrepreneurship. Conservation of resources theory can shed light on the mechanism of entrepreneurial identity affecting entrepreneurial subjective well-being from the perspective of entrepreneurial identity to individual internal resource changes. According to conservation of resources theory, individuals always try to obtain and preserve resources (Hobfoll, 2001). Entrepreneurship mainly answers the question related to “who am I” or “whom I want to be.” During self-identity construction and identification, entrepreneurs may stimulate different identity recognition and emotional experience, which will consume more resources and affect their subjective well-being to varying degrees. Work rumination refers to the state of individuals repeatedly thinking about work-related problems and events outside of work (Querstret and Cropley, 2012), which mainly includes work-related problem-solving pondering and work-related affective rumination (Kinnunen et al., 2017). Positive rumination during off-working hours can effectively predict employees' active behavior and organizational citizenship behavior, which helps improve well-being (Binnewies et al., 2009). Other studies find that high-working ruminant individuals experience more emotional exhaustion (Flaxman et al., 2018) and less well-being (Flaxman et al., 2012). To reveal the impact of entrepreneurial identity on entrepreneurial subjective well-being, based on resource conservation theory, this study uses the two-dimensional structure of work rumination as a mediator to construct a double-edged sword model that affects the subjective well-being of entrepreneurship during the entrepreneurial process. The model includes a “blade of advantage” and a “blade of disadvantage.” The former is the resource gain path in which entrepreneurial identity promotes work-related problem-solving pondering and then affects subjective well-being. The “blade of disadvantage” is the resource depletion path in which entrepreneurial identity induces work-related affective rumination and endangers entrepreneurial subjective well-being. Thus, entrepreneurial identity is likely to be a double-edged sword, which brings benefits and costs to entrepreneurs by inducing different kinds of rumination on their work.

If entrepreneurial identity is a double-edged sword, under what conditions does entrepreneurial identity contribute to entrepreneurial subjective well-being and when does it inhibit it? Only by defining the boundaries of the double-edged sword effect of entrepreneurial identity can we more effectively amplify the positive effect of the “blade of advantage” and avoid the negative effect of the “blade of disadvantage.” Entrepreneurs can then effectively intervene in their cognition and behavior during entrepreneurial practice. The proposed model provides a good formula for the enhancement of entrepreneurial subjective well-being. According to resource conservation theory, under the condition of different individual resources, there are different levels of resource gain and resource depletion caused by individual behavior, which subsequently has different effects on well-being (Hobfoll, 2002). Therefore, in the context of conservation of resources theory, this study introduces individual characteristic variables that are closely related to entrepreneurs' resources, namely, mindfulness. Mindfulness refers to an individual's uncritical attention and awareness of current events and experiences (Glomb et al., 2011). Mindfulness, as an important and unique individual internal resource (Montani et al., 2018; Fisher et al., 2019), shows an individual's specific initial resource state. Different from the psychological capital resources that have been widely discussed in the past (Xanthopoulou et al., 2007; Kang and Peng, 2020), mindfulness is more closely related to how individuals use their attention resources (Grover et al., 2017). Existing research shows that mindfulness enables individuals to observe internal and external stimuli without judgment or evaluation, thereby enhancing their awareness and discovery of resources (Kroon et al., 2015). On the one hand, mindfulness may increase the level of entrepreneurial identity and drive entrepreneurs to actively access internal and external resources, thus facilitating resource gain, such as enhancing problem-solving contemplation. On the other hand, mindfulness may moderate the double-edged sword effect of entrepreneurial identity by replenishing the energy consumed by entrepreneurs' identity and slowing resource depletion, such as reducing emotional rumination.

**Figure 1:**
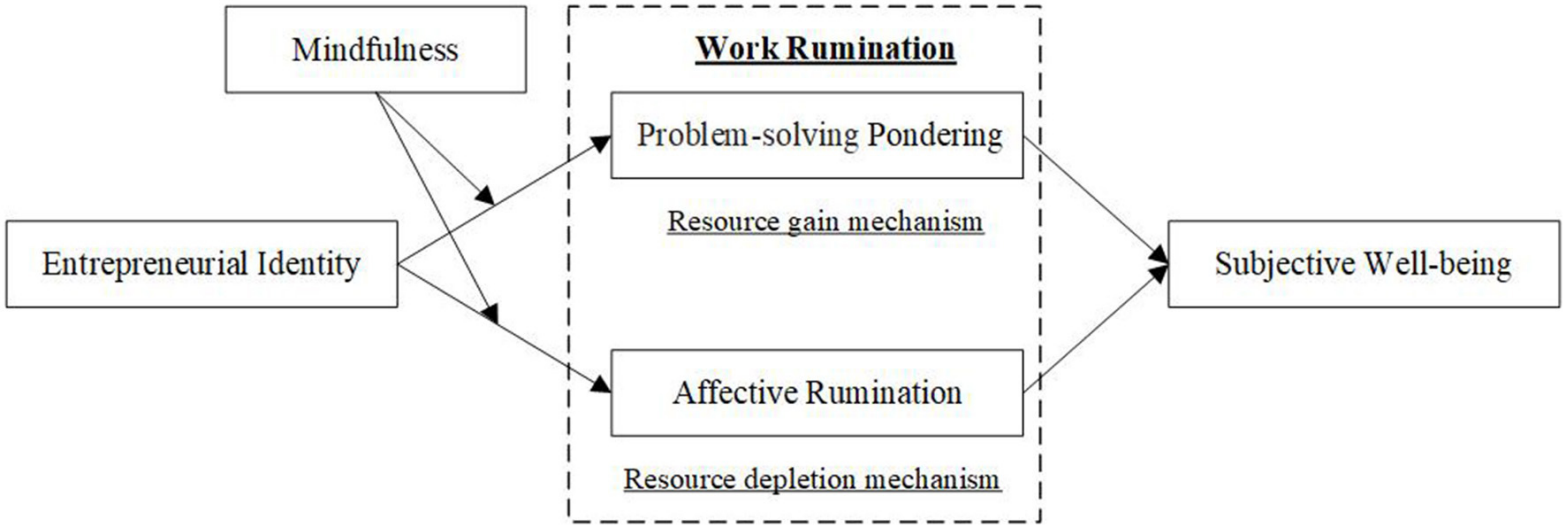
Theoretical model.

Based on the above analysis, this study uses conservation of resources theory to propose a theoretical model that investigates the double-edged sword effect of entrepreneurial identity. This study also uses the two dimensions of work rumination as a mediator and entrepreneurs' mindfulness as a moderating variable, as shown in Figure 1. The contribution of this study is three-fold. First, previous studies on entrepreneurial well-being focus on the “outcome effect” of entrepreneurship. This work reveals the relationship between entrepreneurial identity and entrepreneurial subjective well-being based on the “process effect” of entrepreneurship, clarifies its positive and negative effects, and complements and expands the outcome variable of entrepreneurial identity. Second, previous studies mostly explore the effects of entrepreneurial well-being from the perspectives of difference theory and self-determination theory. Based on conservation of resources theory, this study explores the two paths of resource gain (work-related problem-solving pondering) and resource depletion (work-related affective rumination) to provide a new and comprehensive perspective for entrepreneurs to rationally view the relationship between entrepreneurial identity and entrepreneurial subjective well-being. Third, previous studies focus on individual psychological capital resources, whereas this research focuses on the moderating effect of entrepreneurs' characteristics of mindfulness. Compared with psychological capital, which centers on helping individuals achieve their goals or values, entrepreneurs can also identify what they value and care about with mindfulness. Entrepreneurs with different levels of mindfulness have a great impact on their cognition level and behavioral decision making. Using an entrepreneur's mindfulness as a moderating variable defines the boundary conditions for entrepreneurial identity to influence entrepreneurs' work ruminations. This shows that entrepreneur's mindfulness has a contingency effect in the double-edged sword of entrepreneurial subjective well-being produced by entrepreneurial identity.

## Theoretical Overview and Hypotheses

### Entrepreneurial Identity

As a special kind of social identity, entrepreneurial identity is derived from but distinguished from professional identity. Compared with the definition of professional identity solely based on self-identity theory, entrepreneurial identity is defined more from the perspective of role internalization, socialization, and collective recognition (Chen et al., 2015). Basing on the perspective of role internalization, scholars believe that entrepreneurs have a clear understanding and affirmative evaluation of self-role construction and internalization into self-concept in the entrepreneurial process. Entrepreneurs who internalize entrepreneurial roles will be motivated to do more activities with consistent roles (Hoang and Gimeno, 2010). Based on the perspective of social categorization, individuals first clarify their social positioning and belonging groups through social classification to identify whether they belong to this group and generate identity. Based on the perspective of collective recognition, scholars emphasize that entrepreneurs gain social recognition by constructing their own identities. Given the above definition, entrepreneurial identity includes two main stage characteristics: (1) determining the entrepreneurial identity means that individuals engage in entrepreneurship to confirm the identity of “entrepreneur,” that is, “who I am”; (2) the integration of entrepreneurial identity means that entrepreneurs integrate their cognitive model with entrepreneurial role characteristics, that is, “who I will be.” Following the viewpoint of Hekman et al. (2009), this study defines entrepreneurial identity as the degree of individual recognition and convergence of the characteristics of the entrepreneur. Entrepreneurial identity plays an important role in entrepreneurial activities and the realization of entrepreneurial goals. It has always been a hot topic in the entrepreneurship research field. The impact of entrepreneurial identity is mainly at individual level and venture level. At the individual level, entrepreneurial identity can stimulate entrepreneurial passion (Murnieks et al., 2020), promote opportunity recognition and exploitation (Wry and York, 2017), and enhance entrepreneurial willingness (Mahto and McDowell, 2018) and entrepreneurial behavior (Anderson et al., 2019). At the venture level, entrepreneurial identity can effectively promote external resources acquisition (Wry and York, 2017), increase the creation of entrepreneurial enterprises (Pan et al., 2019), improve entrepreneurial performance (Oo et al., 2019), and promote the growth of enterprises (Ladge et al., 2019). Few scholars empirically examined the relationship between entrepreneurial identity and entrepreneurs' subjective well-being at the individual level.

To theoretically explain the dynamic process of entrepreneurial identity influencing the subjective well-being of entrepreneurship, this study introduces conservation of resources theory as a theoretical lens. Resource conservation theory is one of the strong influential theories in the research explaining the formation of individual well-being (Hobfoll, 2002). According to conservation of resources theory, individuals have limited resources and will continue to invest in resources to protect resources of existing value from loss (Hobfoll, 2011). This may have an important impact on their happiness. Recent research finds that conservation of resources theory has a strong explanatory role in explaining how CEO stewardship affects job well-being. Specifically, CEO stewardship behavior may produce both positive events that increase resources and negative events that deplete resources. The former enhances the CEO's positive emotions and improves well-being, whereas the latter increases the workload and reduces well-being (Kang and Peng, 2020). Thus, this study reveals the double-edged sword effect of entrepreneurial identity on entrepreneurs' subjective well-being based on conservation of resources theory. This effect pertains to the resource gain path that stimulates entrepreneurial subjective well-being and the resource depletion path that induces entrepreneurial subjective well-being.

### Positive Effect of Entrepreneurial Identity on Entrepreneurship Subjective Well-Being: A Resource Gain Path of Work-Related Problem-Solving Pondering

As a state of work rumination, work-related problem-solving pondering reflects an individual's continuous psychological review of specific problems. It is measured by evaluating how previous work has been effectively improved, including thinking about problems from a new perspective, solving existing obstacles, and discovering creative methods. By facilitating problem solving, individuals have positive emotions and thus have a pleasant experience (Cropley and Zijlstra, 2011). According to conservation of resources theory, this study holds that entrepreneurial identity may promote work-related problem-solving pondering. Work-related problem-solving pondering is an individual's purposeful adjustment of cognition. The cognitive needs of entrepreneurs are an important inducement for work-related problem-solving pondering (Wach et al., 2021), and the identity of entrepreneurs can provide entrepreneurs with corresponding cognitive resources. First, entrepreneurial identity is strongly associated with entrepreneurial behavior (Hoang and Gimeno, 2010). Entrepreneurs usually have multiple identities in the entrepreneurial process (Mathias and Williams, 2017). When entrepreneurs assume one identity, they exhibit thinking and actions that distinguish them from other identities (Murnieks et al., 2011). Sometimes though, a series of conflicting behavior expectations need to be dealt with at the same time (Horton et al., 2014). The fact that entrepreneurs play different roles simultaneously further enhances entrepreneurial self-perceived resources. Second, entrepreneurs will inevitably encounter “waterloo” while starting a business. Entrepreneurs' identity may inspire them to maintain their multiple identities, which can not only encourage entrepreneurs to conduct self-verification but also spend more time and energy on role identity management to improve personal cognitive resources. Finally, an uncertain entrepreneurial environment leads to increased entrepreneurial risk, and entrepreneurial identity can lead to a strong sense of security and belonging, enhancing motivation to strive for ideals and inspiring strong entrepreneurial enthusiasm (Murnieks et al., 2014). Passionate entrepreneurs usually show higher entrepreneurial effort in their entrepreneurial activities (Cardon et al., 2017). Entrepreneurs usually actively reflect on their entrepreneurial efforts, actively learn, and constantly try to solve difficult problems and improve the methods and efficiency of their work in entrepreneurship. All these greatly enhance entrepreneurial problem-solving skills. Therefore, entrepreneurial identity may drive entrepreneurs to access appropriate cognitive resources that can effectively contribute to work-related problem-solving pondering. Work-related problem-solving pondering can further influence entrepreneurial well-being (Wach et al., 2021). Resource conservation theory emphasizes that individuals with abundant resources can obtain more resources to achieve resource gain (Zheng et al., 2015). With sufficient cognitive resources, entrepreneurs may promote work-related problem-solving pondering, which helps entrepreneurs solve problems in the entrepreneurial process, thereby enhancing their subjective well-being. Studies confirm that work-related problem-solving pondering has a significant positive effect in the field of work. For example, in the diary study of entrepreneurs, problem-solving pondering can significantly improve work creativity (Weinberger et al., 2018). In addition, problem-solving pondering has a stable cross-time effect in promoting the positive effects of work (Vahle-Hinz et al., 2017; Flaxman et al., 2018). Our research suggests that entrepreneurs' work-related problem-solving pondering will enhance their subjective well-being. The reasons are as follows: First, entrepreneurs always face a high uncertainty when starting a business and often need to solve the emerging difficulties. When entrepreneurs are thinking about a certain problem or discovering a certain abnormal phenomenon, they tend to adjust his cognitive schema to explain the abnormality, consider the rationality of the abnormal phenomenon, and seek more possibilities to solve the problem. This work-related problem-solving pondering may stimulate entrepreneurs' inspiration and thinking. In solving a core problem, the positive experience of problem-solving caused by sudden or unexpected “insights” may trigger entrepreneurs' subjective well-being. Second, entrepreneurs' work-related problem-solving pondering is seen as an experience of resource acquisition, involving emotional neutrality and constructive thinking, which helps to solve related work problems (Cropley and Zijlstra, 2011). When starting a business, entrepreneurs may be interested in how to improve their entrepreneurial performance. For example, when new products are introduced to the market, entrepreneurs may spend more time thinking and looking for business opportunities in their spare time. Through the comparative analysis of different schemes, entrepreneurs will further experience positive emotions and improve subjective well-being through work-related problem-solving pondering. The positive affect that individuals experience every day can significantly increase their daily job satisfaction and work engagement (Koopman et al., 2016). In summary, the identity of entrepreneurs promotes work-related problem-solving pondering, and work-related problem-solving pondering further improves their subjective well-being.

H1: Entrepreneurial identity will promote entrepreneurs' work-related problem-solving pondering and then enhance their subjective well-being.

### Negative Effect of Entrepreneurial Identity on Entrepreneurial Subjective Well-Being: The Resource Depletion Path of Emotional Regurgitation of Work-Related Problems

Work-related affective rumination is a negative cognitive state induced by the obstruction of work-related goals. It is mainly generated by work context and focuses on the negative emotional experience brought by work experience, which is intrusive, extensive, repetitive, and other characteristics (Cropley and Zijlstra, 2011). According to conservation of resources theory, under conditions of limited resources, individuals will invest in resources to protect existing resources from loss and to acquire new resources (Hobfoll, 2011). Although entrepreneurial identity can reap multiple resources, it also requires a drain on personal resources. This study argues that the destroyed effects of entrepreneurial identity may induce work-related affective rumination. The process of entrepreneurial identity is a resource investment and resource acquisition behavior. Entrepreneurs' pursuit of their own identity and the strengthening of their identity may consume their time, physical energy, and attention, and other resources, which in turn can lead to negative emotional experiences such as emotional exhaustion. Individuals are more likely to induce negative emotional experiences when work is characterized by demanding features such as “heavy workload,” “high emotional demands,” and “high conflict decision making” (Demerouti et al., 2001; Bakker et al., 2003). Entrepreneurial identity has exactly these characteristics. First, entrepreneurs have multiple role identities, and in certain contexts, entrepreneurs will extract a particular identity from the “role cluster” to determine their behavioral expectations. This expenditure of more work time and resources to satisfy the self-role identity increases the workload of the entrepreneur. Second, entrepreneurial identity is inseparable from external support and recognition of the entrepreneurial identity role. When building and maintaining relationships and interactions with external stakeholders, entrepreneurs need to control and adjust their emotions to cope with various challenges and obstacles, which makes entrepreneurs more prone to emotional attrition. Finally, when making decisions, entrepreneurs not only consider the role conflicts arising from their multiple identities but also weigh the conflicts of different stakeholders, which can consume plenty of executive resources such as attention and thinking. This then exacerbates entrepreneurs' emotional exhaustion. Entrepreneurial identity requires more work and tasks to be tackled and more time and energy to be spent, which can easily put entrepreneurs in a situation of work-related affective rumination.

Work-related affective rumination affects the well-being of entrepreneurs (Wach et al., 2021). According to conservation of resources theory, when an individual's physical and mental resources are threatened, the pressure experienced will lead to the depletion of resources. As a negative emotional experience, work-related affective rumination can destroy entrepreneurs' physical and mental resources, which can lead to a depletion of resources and then endanger the entrepreneur's subjective well-being. First, in a state of resource depletion, entrepreneurs face more challenges and pressures as the level of emotional regurgitation at work rises, making it difficult for them to achieve their desired goals and reducing their subjective well-being if they are unable to resolve current challenges in a timely manner. Second, individuals with work-related affective rumination tend to view work as an uncontrollable and unavoidable stressor (Pravettoni et al., 2007). This persistent ruminative thinking by entrepreneurs keeps cognitive resources occupied, occasionally depleting resources allocated to other tasks (Connolly et al., 2014). This constant depletion of cognitive resources for negative work and affective experiences may leave individual resources such as guaranteed sleep unrepaired, thus continuing to impair work role performance (Weinberger et al., 2018) and indirectly reducing entrepreneurs' subjective well-being. Finally, individuals with high levels of affective rumination tend to experience more emotional exhaustion (Flaxman et al., 2018), produce more feelings of weariness or spiritless (Querstret and Cropley, 2012), worsen depression (Vandevala et al., 2017), and reduce executive function (Cropley et al., 2016). This negative effect will continuously reduce entrepreneurs' job satisfaction, thereby reducing their subjective well-being. Research on the relationship between emotional rumination and well-being is supported in the previous literature. Entrepreneurial emotional appeals enhance affective rumination under entrepreneurial hindering stressors, thereby reducing entrepreneurial well-being (Wach et al., 2021). In summary, entrepreneurial identity increases work-related affective regurgitation, which in turn worsens their subjective well-being.

H2: Entrepreneurial identity enhances entrepreneurs' work-related affective rumination, which in turn harms their subjective well-being.

### Moderating Role of Mindfulness

Mindfulness refers to an individual's uncritical attention and awareness of current events and experiences (Glomb et al., 2011). According to conservation of resources theory, the level of individual resources will affect the changes of their physical and mental resources in a specific situation. When individuals have rich personal resources, they can effectively make up for the lack of resources; when they have fewer personal resources, they cannot supplement the lack of resources, and it is difficult to obtain other resources (Hobfoll, 2002). As a vital and unique internal resource (Montani et al., 2018; Fisher et al., 2019), mindfulness demonstrates an individual-specific initial resource state. Mindfulness plays a crucial role in individual attitudes and behaviors (Good et al., 2016; Cood and Salanova, 2018). Mindfulness can be effective in soothing individuals' negative emotions, reducing exposure to stress and burnout (Nezlek et al., 2016), improving work performance (Dane and Brummel, 2014; Liu et al., 2020), and enhancing leadership performance (Karssiens et al., 2014). Previous research based on resource conservation theory also validates the moderating effect of mindfulness. For example, Wan et al. (2021) used a sample of 222 working college students in the United States and found that working college students with high levels of mindfulness are able to effectively supplement their resources and mitigate the negative effects of time pressure on work–school conflict. Qian et al. (2020), based on a sample of 161 leader-employee pairs as subjects in China, found that mindfulness is effective in compensating for individuals' psychological resources in the organization and reducing individuals' emotional depletion, thereby mitigating the negative effects of job insecurity on transformational leadership behavior. This suggests that mindfulness has the function of supplementing or increasing one's psychological resources, enhancing the gaining effect of resources, and slowing down the depleting effect of resources. According to resource conservation theory and the literature on mindfulness, we hold that mindfulness can not only enhance the resource level of entrepreneurs' identity, drive entrepreneurs to obtain resources, and promote resource gain (i.e., improve work-related problem-solving pondering) but also slow down the loss of resources consumed by entrepreneurs' identity (i.e., reduce work-related affective rumination).

Conservation of resources theory emphasizes that individuals with abundant resources tend to pursue more resources to enhance resource gain, whereas individuals with few resources avoid more resource loss to reduce resource loss (Hobfoll et al., 1990). Thus, different levels of mindfulness among entrepreneurs may influence their resource status and their sensitivity to resource gain and loss. This study argues that entrepreneurs with high levels of mindfulness may view entrepreneurial identity as an opportunity to access resources, thereby promoting work-related problem-solving pondering. On the one hand, mindful individuals tend to exhibit high levels of self-awareness (Vago and David, 2012). The higher the level of mindfulness of entrepreneurs, the more likely they are to be separated from the past and the future, focusing on the current entrepreneurial identity construction and identification experience. They will be fully committed, remain alert to entrepreneurship, always pay attention to external changes, constantly receive new information, and improve their own experience and capabilities. This present-focused experience and open-ended awareness can provide a positive way for entrepreneurs to cope, especially when faced with entrepreneurial difficulties and setbacks. On the other hand, mindfulness not only helps individuals achieve their goals, as traditional individual resources do but also helps individuals identify what they care about (Shapiro et al., 2006). As entrepreneurs face the process of constructing and identifying with complex role identities, those with high mindfulness may have a better chance of identifying and choosing entrepreneurial role identities that are more authentic and more in line with their values, needs, and interests, and thus continuously self-regulate. On the contrary, entrepreneurs with low mindfulness levels have fewer resources themselves. Under the condition of few resources, entrepreneurs are unlikely to regard identity as access to resources. This restricts entrepreneurs' access to resources through identity, thus hindering the gain of resources. Previous studies also find that under the condition of fewer resources, it is extremely difficult for individuals to try to use existing resources to obtain more additional resource gains (Hobfoll, 2002). Based on the above logic, the following hypotheses are proposed in this study:

H3: Compared with the low level of mindfulness of entrepreneurs, the high level of mindfulness of entrepreneurs has a stronger relationship between entrepreneurial identity and work-related problem-solving pondering.

Although conservation of resources theory emphasizes the “priority of loss,” it also suggests that the positive resources acquired by individuals can effectively supplement the negative effects of resource depletion through resource expenditure (Hobfoll, 2011). When individuals realize that resource consumption in a short period can effectively obtain long-term investment returns, they can alleviate the tension and pressure caused by high work requirements (Halbesleben et al., 2014). Therefore, we believe that entrepreneurs' high level of mindfulness can effectively cope with the multiple pressures faced by entrepreneurs' identity and effectively alleviate the depletion effect of entrepreneurial identity on work-related affective rumination. Specifically, on the one hand, individuals with mindfulness usually have a high capacity for self-regulation (Glomb et al., 2011). This ability to manage and perceive emotions in entrepreneurs with high levels of positive thinking may accommodate and resolve the negative emotional experiences associated with role-identity conflict and work stress, thereby reducing work-related affective rumination. Mindfulness is closely associated with emotions and that mindfulness can lead individuals to accurately and effectively perceive and regulate their emotional states, helping them to reduce overreaction and emotional dysregulation and achieve a state of good emotional regulation (Good et al., 2016). On the other hand, individuals with high mindfulness rely on open and flexible thought processing and openness to internal experiences and external stimuli (Glomb et al., 2011). Employees with high mindfulness can achieve positive assessments to view their emotions in a clearer and more objective way by tuning their self-attention (Hülsheger et al., 2013). In short, when they experience negative emotions, they do not get caught up in it. Therefore, individuals with high mindfulness can be separated from negative emotional states to repair their negative emotions. This objective acceptance enhances individuals' self-awareness of distinguishing events induced by thoughts or emotions, thereby enhancing their grasp of the environment and self-control (Zhang et al., 2017). The uncritical acceptance of entrepreneurs with high levels of mindfulness may better slow down work-related affective rumination. By contrast, entrepreneurs with low levels of mindfulness are more likely to fall into the work-related affective rumination dilemma due to the lack of sufficient external resources to compensate for the loss of entrepreneurial identity. Furthermore, due to limited individual resources, entrepreneurs with low levels of mindfulness may value the depletion of resources more, which further increases work-related affective rumination perceptions and traps them in a negative emotional dilemma. Low psychological capital not only fails to compensate for lost personal resources from workload but also fails to enhance resource gains in positive emotions for the CEO, which in turn endanger the CEO's own well-being (Kang and Peng, 2020). Recent research confirms the buffering effect of mindfulness on the relationship between job insecurity and emotional exhaustion (Qian et al., 2020). Based on the above discussion, we proposed the following hypothesis:

H4: Compared with the low level of mindfulness of entrepreneurs, the high level of mindfulness of entrepreneurs has a weaker relationship between entrepreneurial identity and work-related affective rumination.

## Methods

### Participants and Procedures

This study mainly adopts the questionnaire survey method and relies on the Wenjuanxing platform (https://www.wjx.cn) to collect online questionnaires data from entrepreneurs in cities across the country, involving IT, education, retail, and other industries. Taking part-time or graduated MBA students from the business college as the research object, through snowballing method, we asked them to invite their partners who are starting their own business or have experience in starting a business around them to participate in our research. The whole research process is roughly divided into three steps. First, the group members counted the information about MBA students who had entrepreneurial experience or starting a business. Second, the research team members sent the valid link address of the electronic questionnaire to the respondents and asked them to invite their business partners to fill in the questionnaire. Finally, after the questionnaires were collected, the researchers coded, screened, and sorted the questionnaires. The entire survey lasted nearly 1 week, and a total of 1,411 questionnaires were collected. After excluding invalid samples, a total of 882 valid samples were finally obtained, with an effective recovery rate of 62.5%. The descriptive characteristics of the samples are shown in Table 1.

**Table 1:** Descriptive characteristics of samples (*N* = 882).

**Characteristic**	**Category**	**Number**	**Percentage(%)**	**Characteristic**	**Number**	**Number**	**Percentage(%)**
Gender				Age	<25 years	272	30.8
	Male	480	54.4		26~30 years	313	35.5
	Female	402	45.6		31~35 years	208	23.6
					>36 years	89	10.1
Education level	High school degree or below	60	6.8	Start-up business years	≤ 2 years	203	23
	Junior college degree	379	43		3~5 years	472	53.5
	Bachelor degree	342	38.8		>5 years	207	23.5
	Master degree or above	101	11.5				
Number of entrepreneurs	≤ 5	110	12.5	Industry	Entertainment industry	256	29
	5~20	337	38.2		IT industry	341	38.7
	21~40	325	36.8		Education industry	128	14.5
	≥41	110	12.5		Retail industry	109	12.4
					Other industry	48	5.4

### Measures

The measurement scales used in this study were all based on well-established scales with high reliability and validity. To ensure the quality of the questionnaire, we carried out back-translation on all English scales, using seven-point Likert ranging from 1 (totally disagree) to 7 (completely agree). All scale items used in our research can be found in Appendix A.

### Entrepreneurial Identity

Based on the viewpoint of Hekman et al. (2009), this study defines the entrepreneurial identity as the degree of individual recognition and convergence of the characteristics of the entrepreneur. The measurement of the independent variable entrepreneurial identity was measured with a six-item scale developed by Hekman et al. (2009). Sample items include “In general, when someone praises entrepreneurs, it feels like a personal compliment.” The Cronbach's α is 0.965, and the composite reliability is 0.966.

### Work Rumination

Work rumination refers to the state of individuals repeatedly thinking about work-related problems and events outside of work (Querstret and Cropley, 2012). We adopted a 10-item Work-Related Rumination Questionnaire (WRRQ) to measure two different kinds of work rumination (Cropley et al., 2012; Querstret and Cropley, 2012). Work-related problem-solving pondering has five items. An example is “After work I tend to think of how I can improve my work-related performance.” The Cronbach's α was 0.976, and the composite reliability is 0.9764. Work-related affective rumination has five items. An example is “I become tense when I think about work-related issues during my free time.” The Cronbach's α is 0.951, and the composite reliability is 0.9523.

### Mindfulness

Mindfulness refers to an individual's uncritical attention and awareness of current events and experiences (Glomb et al., 2011). The moderator variable mindfulness was measured with a five-item scale from Hülsheger et al. (2013). Sample items include “I found it difficult to stay focused on what was happening in the present.” The Cronbach's α is 0.980, and the composite reliability is 0.9804.

### Entrepreneurs' Subjective Well-Being

Based on the viewpoint of Newman et al. (2018), this study defines entrepreneurs' subjective well-being as an entrepreneur's overall assessment and subjective feelings about his or her quality of life, job satisfaction, and personal growth and development during the creation and operation of the business. The dependent variable entrepreneurs' subjective well-being was measured using a five-item scale from Newman et al. (2018). Sample items include “In most ways my life is close to my ideal.” The Cronbach's α is 0.961, and the composite reliability is 0.9611.

### Control Variables

Seeing that we based our research on resource conservation theory to examine how entrepreneurial identity affect entrepreneurs' subjective well-being, we referred to previous studies on entrepreneurs' well-being. We took the entrepreneur's gender, age, education level, years of entrepreneurship, number of entrepreneurs, and industry as control variables.

## Results

### Common Method Bias Analyses

The variables involved in this study are all self-reported by entrepreneurs, which may incur the issues of common method variance. For this reason, we adopted Harman's single-factor test and carried out principal component analysis on all variable measurement items involved in this study. The results show that among the five principal components extracted by the unrotated component matrix, the first principal component with the largest eigenvalue explains the variance of 42.824%, less than half of the total variance of 87.901%, which shows no obvious common method variance in this study.

### Confirmatory Factor Analyses

In our study, confirmatory factor analysis is used to examine the distinguishing validity of entrepreneurial identity, work-related problem-solving pondering, work-related affective rumination, mindfulness, and entrepreneurial subjective well-being. As shown in Table 2, a five-factor model fits the data better (χ2/df = 1.304, CFI = 0.997, TLI = 0.997, RMSEA = 0.019) than the other factor models such as the four-factor, three-factor, and so on, which indicates that the variables have good discriminant validity and can represent the four different constructs.

**Table 2:** Confirmatory factory analyses.

**Model**	*****χ^2^*****	***df***	*****χ^2^**/df***	***Δχ2(Δdf)***	***CFI***	***TLI***	***RMSEA***
Five-factor model:EI, WPP, WAR, MIF, SWB	376.886	289	1.304	–	0.997	0.997	0.019
Four-factor model:EI, WPP + WAR, MIF, SWB	4,980.399	293	16.998	4,603.513^***^(4)	0.851	0.834	0.135
Three-factor model:EI, WPP + WAR + MIF, SWB	10,691.798	296	36.121	10,314.912^***^(7)	0.669	0.636	0.200
Two-factor model:EI+WPP + WAR + MIF, SWB	16,110.227	298	54.061	15,733.341^***^(9)	0.450	0.496	0.245
One-factor model:EI+WPP + WAR + MIF + SWB	20,582.986	299	68.839	20,206.100^***^(10)	0.353	0.297	0.277

*N = 882*,

*** *p < 0.001. EI, Entrepreneurial Identity; WPP, Work-related Problem-solving Pondering; WAR, Work-related Problem-solving Pondering; MIF, Work-related Affective Rumination; SWB, Subjective Well-being. “+” means Integration; CFI, Comparative Fit Index; TLI, Tucker-Lewis Index; RMSEA, Root-Mean-Square Error of Approximation*.

### Preliminary Analyses

Table 3 provides the means, standard deviations, and correlation coefficients for each variable. The results are shown in Table 3, where work-related problem-solving pondering is significantly positively related to entrepreneurial identity (*r* = *0.594, p* < *0.01*) and entrepreneurial subjective well-being (*r* = *0.402, p* < *0.01*), H1 is therefore preliminarily supported. Moreover, work-related affective rumination is significantly positively related to entrepreneurial identity (*r* = *0.095, p* < *0.01*), and to entrepreneurial subjective well-being (*r* = −*0.107, p* < *0.01*) is significantly negatively related to entrepreneurial subjective well-being (*r* = –*0.107, p* < *0.01*), H2 is therefore preliminarily supported. Mindfulness is significantly positively related to entrepreneurial identity (*r* = *0.505, p* < *0.01*) and work-related problem-solving rumination (*r* = *0.523, p* < *0.01*), suggesting that mindfulness may have a positive effect on work-related problem-solving pondering. Mindfulness is positively related to work-related affective rumination (*r* = –*0.81, p* < *0.01*) and significantly negatively correlated, suggesting that mindfulness may harm work-related affective rumination. In addition, work-related problem-solving pondering is not significantly correlated with work-related affective rumination *(r* = *0.024, p* > *0.05*).

**Table 3:** Mean, Standard deviations, and correlations for all variables (*N* = 882).

**Variables**	**Mean**	**SD**	**1**	**2**	**3**	**4**	**5**	**6**	**7**	**8**	**9**	**10**	**11**
1. Gender	0.540	0.498	—										
2. Age	2.130	0.966	−0.019	—									
3. Education	2.550	0.783	0.005	−0.047	—								
4. Establishment	2.000	0.682	0.033	0.054	−0.013	—							
5. Number	2.490	0.866	0.022	0.039	−0.003	0.817^**^	—						
6. Industry	2.270	1.162	−0.014	0.049	−0.009	−0.030	−0.031	—					
7. EI	4.863	1.476	−0.056	−0.059	−0.007	0.048	0.062	−0.032	**0.826**				
8. WPP	4.878	1.531	−0.022	−0.089^**^	−0.067^*^	0.080^*^	0.060	−0.011	0.594^**^	**0.892**			
9. WAR	4.734	1.060	−0.029	0.041	0.008	0.023	0.006	0.003	0.095^**^	0.024	**0.800**		
10. MIF	3.969	1.713	−0.025	−0.092^**^	−0.043^*^	0.05	0.070^*^	0.027	0.505^**^	0.523^**^	−0.081^*^	**0.909**	
11. SWB	4.781	1.219	0.028	−0.043	−0.049	0.043	0.026	0.001	0.402^**^	0.383^**^	−0.107^**^	0.286^**^	**0.832**

* *p < 0.05*,

** *p < 0.01. Entry on the diagonal is the square roots of average variances extracted (AVE). Bold values indicate the square roots of average variances extracted, which is a statistic for testing the internal consistency of structural variables*.

### Analyses of the Main Effect and Mediating Effect

Following the test steps of the mediation effect proposed by Baron and Kenny (1986), this study uses hierarchical regression analysis to verify whether work-related problem-solving pondering and work-related affective rumination have a mediating effect between entrepreneur's identity and entrepreneurial subjective well-being. The analysis results are given in Table 4. Regarding the mediating effect of work-related problem-solving pondering, the specific results are shown in Table 4. After controlling the statistical characteristics of entrepreneurs, entrepreneurial identity has a significant positive impact on entrepreneurial subjective well-being (*Model 6*, β = *0.333, p* < *0.001*), and entrepreneurial identity has a significant positive effect on work-related problem-solving pondering (*Model 2*, β = *0.612, p* < *0.001*). Model 7 shows a significant positive effect of work-related problem-solving pondering on entrepreneurial subjective well-being (β = *0.303, p* < *0.001*) after controlling for individual statistical characteristics of the entrepreneurs. When both the independent and mediating variables are included in the regression equation model, a significant positive effect of work-related problem-solving pondering on entrepreneurial subjective well-being remains (*Model 9*, β = *0.172, p* < *0.001*). The effect of entrepreneurial identity on entrepreneurial subjective well-being remains significant, but the regression coefficient changed from β = *0.333* (*p* < *0.001*) to β = *0.228 (p* < *0.001)*. It can be concluded that work-related problem-solving pondering partially mediates the relationship between entrepreneurial identity and subjective entrepreneurial well-being. Thus, H1 is supported. As for the mediating effect test of work-related affective rumination, as can be seen from Table 4, entrepreneurs' identity has a significant positive effect on work-related affective rumination (*Model 4*, β = *0.069, p* < *0.05*), and work-related affective rumination has a significant negative effect on entrepreneurial subjective well-being (*Model 8*, β = −*0.122, p* < *0.001*). When the independent and mediating variables are included in the regression equation model, a significant negative effect of work-related affective rumination on entrepreneurial subjective well-being is still noted (*Model 10*, β = −*0.168, p* < *0.001*). The influence of entrepreneurial identity on entrepreneurial subjective well-being remains significant, and the coefficient changes from β = 0.333 (*p* < *0.001*) to β = 0.345 (*p* < *0.001*). However, the indirect effect of work-related affective rumination is negative. The regression coefficient of entrepreneurial identity and work-related affective rumination and the regression coefficient of work-related affective rumination and entrepreneurial subjective well-being are different. The regression coefficients of main effects and indirect effects have opposite signs. According to the judgment method of “mediating effect” and “suppressing effect” by Wen and Ye (2014), work-related affective rumination is not a partial mediating effect between entrepreneurial identity and entrepreneurial well-being, but a partial “suppressing effect.” On the one hand, the existence of the “suppressing effect” of work-related affective rumination shows that the entrepreneurial identity indirectly affects the subjective well-being of entrepreneurs through work-related affective rumination. On the other hand, it also indicates a more effective intermediary variable between independent and dependent variables (Kenny et al., 2003). There is still a strong intermediary mechanism between entrepreneurial identity and entrepreneurial subjective well-being, which is not included in the vision of this study and, thus, needs to be further explored in the follow-up research.

**Table 4:** The mediation effect test of work rumination.

**Variable**	**WPP**		**WAR**		**SWB**					
	**Model 1**	**Model 2**	**Model 3**	**Model 4**	**Model 5**	**Model 6**	**Model 7**	**Model 8**	**Model 9**	**Model 10**
Gender	−0.081 (0.103)	0.025 (0.083)	−0.063 (0.072)	−0.051 (0.072)	0.063 (0.082)	0.121 (0.076)	0.088 (0.076)	0.056 (0.082)	0.117 (0.074)	0.113 (0.075)
Age	−0.154^**^ (0.053)	−0.097^*^ (0.043)	0.044 (0.037)	0.05 (0.037)	−0.06 (0.043)	−0.029 (0.039)	−0.013 (0.040)	−0.054 (0.043)	−0.012 (0.039)	−0.02 (0.039)
Education	−0.137^*^ (0.065)	−0.126^*^ (0.053)	0.014 (0.046)	0.015 (0.046)	−0.079 (0.053)	−0.073 (0.048)	−0.038 (0.049)	−0.077 (0.052)	−0.051 (0.047)	−0.07 (0.048)
Establishment	0.221 (0.130)	0.224^*^ (0.105)	0.084 (0.091)	0.085 (0.091)	0.115 (0.104)	0.117 (0.096)	0.049 (0.097)	0.126 (0.104)	0.079 (0.094)	0.131 (0.095)
Number	−0.03 (0.103)	−0.099 (0.083)	−0.048 (0.072)	−0.056 (0.071)	−0.035 (0.082)	−0.073 (0.075)	−0.026 (0.076)	−0.041 (0.082)	−0.056 (0.074)	−0.083 (0.074)
Industry	−0.006 (0.044)	0.016 (0.036)	0.001 (0.031)	0.004 (0.031)	0.004 (0.004)	0.016 (0.032)	0.006 (0.033)	0.004 (0.035)	0.013 (0.032)	0.017 (0.032)
EI		0.612^***^ (0.028)		0.069^**^ (0.024)		0.333^***^ (0.026)			0.228^***^ (0.031)	0.345^***^ (0.025)
WPP							0.303^***^ (0.025)		0.172^***^ (0.030)	
WAR								−0.122^**^ (0.039)		−0.168^***^ (0.035)
Intercept	5.245 (0.285)	2.18 (0.270)	4.588 (0.199)	4.24 (0.233)	4.923 (0.229)	3.252 (0.246)	3.335 (0.249)	5.481 (0.288)	2.877 (0.250)	3.963 (0.285)
*R* ^2^	0.021	0.364	0.004	0.013	0.007	0.168	0.149	0.019	0.198	0.189
Δ*R*^2^	–	0.343	–	0.009	–	0.161	0.142	0.012	0.191	0.182
*F*	3.097^**^	71.583^***^	0.529	1.626	1.083	25.290^***^	21.852^***^	2.357^*^	26.941^***^	25.505^***^

*Reported coefficients are unstandardized, standard errors in parentheses*.

* *p < 0.05*,

** *p < 0.01*,

*** *p < 0.001*.

### Analyses of the Moderating Effect

This study uses hierarchical regression to examine the moderating effects of mindfulness on the relationship between entrepreneurial identity and work-related problem-solving pondering and the moderating effects of mindfulness on the relationship between entrepreneurial identity and work-related affective rumination. The analysis results are shown in Table 5. As seen from Table 5, in Models 12 and 14, the values of variance inflation factor are close to one, which indicates no multicollinearity problem among the variables in our study. The values of Durbin–Watson are close to two, which shows no autocorrelation problem among the variables. From Model 12 in Table 5, the interaction between entrepreneurial identity and mindfulness does not have a significant positive effect on work problem-solving pondering (*Model 12*, β = −*0.071, p* > *0.05*), indicating that entrepreneurial mindfulness does not play a positive moderating role between entrepreneurial identity and work problem-solving pondering. H3 is not supported by the findings. The interaction between entrepreneurial identity and mindfulness has a significant negative effect on work-related affective rumination, as seen in Model 14 in Table 5 (*Model 14*, β = −*0.104, p* < *0.01*). This suggests that entrepreneurial mindfulness negatively moderates the relationship between entrepreneurial identity and work-related affective rumination, verifying H4.

**Table 5:** The moderating effect of mindfulness.

**Variable**	**WPP**		**VIF**	**WAR**		**VIF**
	**Model 11**	**Model 12**		**Model 13**	**Model 14**	
Gender	−0.081 (0.103)	0.027 (0.079)	1.006	−0.063 (0.072)	−0.046 (0.071)	1.006
Age	−0.154^**^ (0.053)	−0.061 (0.041)	1.022	0.044 (0.037)	0.043 (0.037)	1.022
Education	−0.137^*^ (0.065)	−0.101^*^ (0.050)	1.005	0.014 (0.046)	0.006 (0.045)	1.005
Establishment	0.221 (0.130)	0.224^*^ (0.100)	3.019	0.084 (0.091)	0.069 (0.089)	3.019
Number	−0.03 (0.103)	−0.119 (0.079)	3.023	−0.048 (0.072)	−0.034 (0.071)	3.023
Industry	−0.006 (0.044)	−0.002 (0.034)	1.008	0.001 (0.031)	0.012 (0.030)	1.008
EI		0.432^***^ (0.035)	1.697		0.091^**^ (0.031)	1.697
MIF		0.280^***^ (0.028)	1.544		−0.081^**^ (0.025)	1.544
EI × MIF		−0.071 (0.041)	1.282		−0.104^**^ (0.037)	1.282
Intercept	5.245 (0.285)	1.927 (0.263)	Durbin-Watson	4.588 (0.199)	4.500 (0.236)	Durbin-Watson
R^2^	0.021	0.430	1.858	0.004	0.043	1.975
ΔR^2^	–	0.409		–	0.039	
*F*	3.097^**^	73.050^***^		0.529	4.377^***^	

*Reported coefficients are unstandardized, standard errors in parentheses*.

* *p < 0.05*,

** *p < 0.01*,

*** *p < 0.001*.

**Figure 2:**
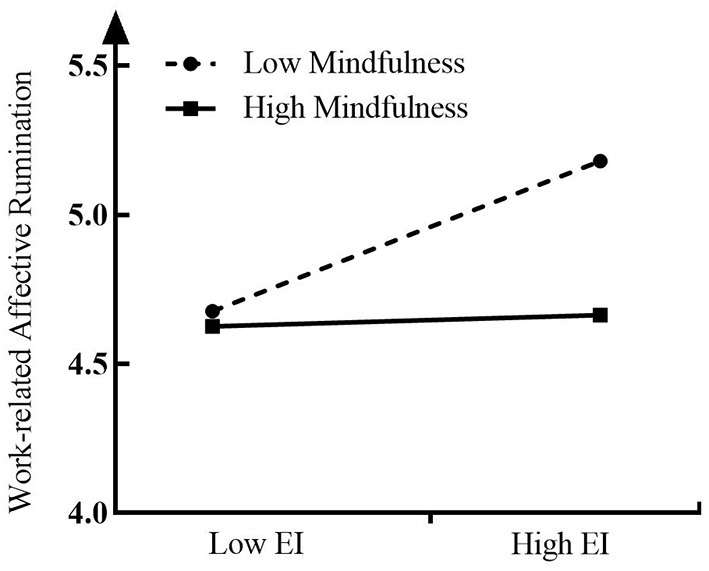
Interactive effect of the entrepreneurial identity and entrepreneurial mindfulness on work-related affective rumination.

To further test whether the moderating effect of entrepreneurial mindfulness on the relationship between entrepreneurial identity and work-related affective rumination is as hypothesized, this study follows the method proposed by Aiken and West (1991) and further conducts a simple slope analysis (seen in Figure 2). Figure 2 shows that when mindfulness is low (M – SD), entrepreneurial identity has a positive effect on work-related affective rumination (γ = *0.171, t* = *4.921, p* < *0.001*), whereas for higher mindfulness (M + SD), entrepreneurial identity is no longer significant on work-related affective rumination (γ = *0.013, t* = *0.256, p* > *0.05*). This finding further supports H4.

## Discussion

Based on conservation of resources theory, this study uses the two dimensions of work rumination as mediating variables to explore the dual effect of entrepreneurial identity on entrepreneurial subjective well-being. This study also examines the moderating effect of mindfulness between entrepreneurial identity and work rumination. Through the empirical test conducted on 882 Chinese entrepreneurs, this research mainly draws the following conclusions. First, entrepreneurial identity has a double-edged sword effect on entrepreneurial subjective well-being. On the one hand, entrepreneurial identity enhances entrepreneurs' subjective well-being by stimulating work-related problem-solving ponding. In the previous research, the positive effect of problem-solving ponding is mainly discussed in the field of work. However, a consistent conclusion on the relationship between problem-solving ponding and subjective well-being has yet to be formed. For example, Querstret and Cropley (2012) found that problem-solving ponding can reduce individual fatigue during the working day. Some studies also shows that problem-solving ponding has no significant relationship with variables in the field of well-being, such as depression experience (Vandevala et al., 2017). Our research shows that problem-solving ponding plays a mediating role in the relationship between entrepreneurial identity and entrepreneurial subjective well-being. This result suggests that entrepreneurs' identity can trigger their problem-solving ponding, which helps produce positive cognitive experiences to enhance their subjective well-being. On the other hand, entrepreneurial identity reduces entrepreneurs' subjective well-being by inducing work-related affective rumination. The harmful effects of affective rumination on variables related to well-being are verified (Wach et al., 2021). For example, individuals with high affective rumination experience more fatigue (Querstret and Cropley, 2012) and are less subjective well-being (Flaxman et al., 2012). Consistent with the above research conclusions, our research finds that entrepreneurs' work-related affective rumination negatively affects entrepreneurial subjective well-being. It is particularly important to note that in the path of resource depletion, work-related affective rumination produces “suppressing effects” between entrepreneurs' identity and entrepreneurial happiness in our study. With the addition of the mediator variable work-related affective rumination, the effect outcome of the influence of entrepreneurial identity on the subjective well-being of entrepreneurship increases instead. The existence of the “suppressing effect” of work-related affective rumination, on the one hand, shows that the mechanism of the relationship between entrepreneurial identity and entrepreneurial subjective well-being does exist. On the other hand, it also indicates that there may be some other mediator variables which need to be further explored.

Second, entrepreneurs' mindfulness does not strengthen the gain of resources but weakens the depletion of resources. This study reveals that the lower an entrepreneur's level of mindfulness, the more entrepreneurial identity is exacerbated by work affective rumination. This finding suggests that entrepreneurs' mindfulness can reduce the cost of entrepreneurial identity. Previous studies present similar findings. When CEOs with low psychological capital lack additional resources to supplement the lack of resources caused by engaging in stewardship behavior, they are more likely to fall into the dilemma of resource depletion and increase their perception of workload (Kang and Peng, 2020). Compared with psychological capital, mindfulness, as an important individual trait, reflects the original and objective state of individual specific resources. When entrepreneurs with a low level of mindfulness lack additional resources to meet the resources required by entrepreneurs' identity, they will fall into a resource dilemma, leading to emotional ruminations related to work. Unfortunately, for highly mindful entrepreneurs, their entrepreneurial identity does not increase work-related problem-solving pondering. The hypothesis regarding the moderating effect of mindfulness between entrepreneurial identity and work-related problem-solving pondering is not supported. We think the possible reason is that entrepreneurs with high levels of mindfulness are less sensitive to resource loss than entrepreneurs with low levels of mindfulness. Work-related problem-solving ponding reflects the individual's purposeful adjustment of self-cognition and self-management, whereas mindfulness emphasizes that the individual maintains uncritical attention and awareness of current events and experiences. Therefore, even when the role of entrepreneurs is threatened, such as entrepreneurial failure, entrepreneurs with a high level of mindfulness are more inclined to keep calmly focusing and thinking, which allows them to recover from a negative emotional state. Mindfulness can also effectively reduce entrepreneurs' excessive attention to negative emotions, allowing them to obtain valuable information from failure events, and weaken the harmful effects of negative emotions on failed learning (Shepherd and Cardon, 2009). It may be for this reason that the moderating role of mindfulness is verified in our study. In addition, when entrepreneurs' identity requires more resources or encounters threats, those with a low level of mindfulness are more likely to be stimulated. It is consistent with the “loss first” principle of resource conservation theory.

### Theoretical Contributions

First, we enrich the research on the influence of entrepreneurial identity on entrepreneurial subjective well-being and expand the antecedent variables of entrepreneurs' subjective well-being. Compared with the psychological well-being generated by the realization of an individual's self-potential, the subjective well-being caused by the individual's emotional experience and life evaluation may be more common given the greater emphasis on the conditions and process of the realization of entrepreneurial results, especially as high uncertainty and high risk are more prominent in the entrepreneurial context. Therefore, the dependent variable of this study is entrepreneurial subjective well-being. Although previous studies explore the antecedent variables of entrepreneurs' subjective well-being from factors such as individual characteristics (Hmieleski and Sheppard, 2019), motivations (Kibler et al., 2019; Amorós et al., 2021), and social networks (Newman et al., 2018; Wu and Song, 2019), as a kind of self-recognition of entrepreneurs, the influence of entrepreneurial identity on entrepreneurs' subjective well-being remains unknown. Previous studies emphasize the positive role of entrepreneurial identity in entrepreneurial decision-making and entrepreneurial performance, but they relatively neglect its potential unfavorable effects. Therefore, this research focuses on the double-edged sword effect of entrepreneurial identity on entrepreneurs' subjective well-being. The results show that entrepreneurial identity can not only promote entrepreneurs' problem-solving pondering to enhance entrepreneurial subjective well-being but also lead to their work-related affective rumination to reduce entrepreneurial subjective well-being. Our research not only enriches the influence of entrepreneurial identity on entrepreneurial subjective well-being but also expands antecedent variables of entrepreneurial subjective well-being.

Second, based on conservation of resources theory, the two-dimensional structure of work ruminating is introduced as mediator variables to integrate the formation mechanism of entrepreneurs' subjective well-being. Existing studies explain the formation of entrepreneurs' subjective well-being based on difference theory and self-determination theory, but these studies mainly focus on entrepreneurial results in entrepreneurial motivation, resource acquisition, and entrepreneurial returns. The “process effect” of entrepreneurship lacks scholarly attention. The entrepreneurship process effect emphasizes that when entrepreneurs value the results, they should also pay attention to the conditions and processes of achieving results. Although some scholars study the formation of entrepreneurial subjective well-being based on resource conservation theory, they only investigate from a single gain mechanism and lack an integrated perspective. For example, Marshall et al. (2020) found that access to entrepreneurial resources can affect entrepreneurs' subjective well-being by the resource acquisition mechanism of entrepreneurial self-efficacy. However, this study does not reveal the depletion mechanism of entrepreneurs' cognitive or behavior on entrepreneurial subjective well-being. Similarly, the conclusion of Leung et al. (2020) shows that emotional and instrumental family support can influence the subjective well-being of SME employers by work–family balance. However, they also did not examine the depletion mechanisms that affect subjective well-being. Based on resource conservation theory, this study integrates resource gain and resource depletion mechanisms to reveal that entrepreneurial identity has both resource gain and resource loss effects by the two-dimensional structure of work rumination as mediator variables. This research enriches the mechanism of entrepreneurial identity influence entrepreneurial subjective well-being and provides new theoretical support for a comprehensive explanation of why an entrepreneur's identity has a dual effect on entrepreneurial subjective well-being. It is helpful for us to deepen the understanding of how entrepreneurial identity affects entrepreneurial subjective well-being.

Third, our research defines the boundary conditions of entrepreneurial identity affecting work-related emotional rumination. In previous studies about work rumination, scholars call for more research on the boundary conditions for the double-edged sword effect of work rumination (Zhang et al., 2020). In this research, under the framework of resource preservation theory, we introduce individual characteristic variables—mindfulness—to further explore its moderating role in the process of entrepreneurial identity influencing work rumination. Our research shows that mindfulness negatively moderates the positive relationship between entrepreneurial identity and work-related affective rumination. In other words, entrepreneurs with a high level of mindfulness can reduce the intensity of entrepreneurial identity rumination against work-related affective rumination. This conclusion suggests that mindfulness can reduce the resource depletion of affective rumination caused by entrepreneurs' identities. Our study extends the existing literature on the boundary conditions of entrepreneurial identity affecting work affective rumination, which helps to provide a clearer understanding of the outcomes of entrepreneurial identity. Additionally, this study helps enrich the application of resource conservation theory from the perspective of individual characteristics.

### Practical Implications

This study finds that the entrepreneurial identity can generate problem-solving pondering related to work, but it may also cause them to fall into affective rumination at work, which reflects a double-edged sword effect. Thus, when enhancing entrepreneurial identity, entrepreneurs should be alert and aware of the potential damage effects of entrepreneurial identity. The conclusions of this research provide theoretical guidance and practical implications for entrepreneurs and policymakers on how to improve entrepreneurs' subjective well-being in entrepreneurship. First, entrepreneurs should take a rational view of entrepreneurial identity because it can trigger two completely different kinds of work rumination, which in turn bring some benefits and costs to happy entrepreneurs. In entrepreneurial practice, even if entrepreneurial identity can play a positive role for entrepreneurs, entrepreneurs should not pursue entrepreneurial identity excessively and unilaterally. Second, entrepreneurs should strengthen their self-management of entrepreneurial subjective well-being. Compared with the cognitive and emotional experience of entrepreneurs, although the entrepreneurial environment and the characteristics of entrepreneurial activities can enhance or inhibit the subjective well-being of entrepreneurs, these factors are often external, and the critical role depends on the entrepreneur himself. Our study finds that entrepreneurs' work-related problem-solving pondering promotes their subjective well-being, but work affective rumination inhibits it. Entrepreneurs should not only improve their ponding ability to solve work problem but also reduce getting bogged down by work-related affective rumination. Entrepreneurs can enhance cooperation and communication among members by entrepreneurial team skills training to improve their cognitive resilience in the thinking process of dealing with problems, especially the core ability to cope with entrepreneurial uncertainty and high failure rate, relieve negative emotional experiences and improve entrepreneurial well-being. Finally, entrepreneurs should focus on cultivating or improving their mindfulness level. Mindfulness has the characteristic of plasticity. Through a series of mindfulness training, entrepreneurs can improve their open-mindedness to new things and maintain stable attention so that they can view problems more objectively and neutrally, thus weakening the negative effect of entrepreneurial identity on work-related affective rumination.

The conclusions of this study guide entrepreneurial policymakers to formulate and implement entrepreneurial policies. First, entrepreneurship policymakers should design flexible management policies to help entrepreneurs handle their daily work arrangements flexibly and enhance the controllability of their own entrepreneurial identity. For example, by adopting a flexible working system, entrepreneurs can effectively respond to the needs of multiple entrepreneurial roles and reduce the possibility of work-related affective rumination. Second, entrepreneurial policymakers can set up entrepreneur training and management projects. The establishment of training management programs can effectively provide entrepreneurs with intellectual support and enhance their identity to obtain scarce external resources that are helpful for entrepreneurship. At the same time, corresponding measures ought to be taken to reduce the potential destroyed effects of entrepreneurial identity. For example, entrepreneurial policymakers can cultivate entrepreneurial incubators, build an online entrepreneurial communication platform, actively build and maintain the resources of entrepreneurs, and create a better entrepreneurial environment for entrepreneurs. It is helpful for entrepreneurs to alleviate the resource depletion caused by entrepreneurial identity, which in turn enhances entrepreneurs' subjective well-being. Finally, entrepreneurial policymakers should improve the entrepreneurial institutions. The entrepreneurial environment has the characteristics of policy variability, discontinuity, and high uncertainty. Only through a healthy institutional environment can entrepreneurs experience entrepreneurial satisfaction and achievement while truly experiencing a fair and competitive market environment. It is helpful for entrepreneurs who are under more pressure and responsibilities, particularly those who are experiencing or preparing to start a business, to truly and fully understand themselves and experience happiness during entrepreneurship.

### Limitations and Research

This study has certain limitations. First, all variables are measured by entrepreneurs' self-assessment at the same point in time, which are cross-sectional data, reflecting the correlation between variables rather than causality. Thus, the causal links among the variables cannot be strictly and precisely tested. Future studies can use longitudinal surveys and experiencing sampling methods to examine the research variables and further improve the accuracy of the research result. Second, based on resource conservation theory, this study proposes and tests a mediating effect of work-related problem-solving pondering and work-related affective rumination. However, work-related affective rumination produces partial “suppressing effects” on the relationship between entrepreneurial identity and entrepreneurial well-being. Although this shows that entrepreneurial identity indirectly affects entrepreneurs' subjective well-being through work-related affective rumination, it also shows that there are mediator variables with greater effects between independent variables and dependent variables (Kenny et al., 2003). Other mediator variables endanger the subjective well-being of entrepreneurship through resource depletion path. For example, individuals exhibit emotional exhaustion that depletes their emotional resources, potentially increasing entrepreneurial ego depletion and thus reducing entrepreneurial subjective well-being. Subsequent research can further examine the mechanisms by which entrepreneurial emotional exhaustion mediates entrepreneurial identity and entrepreneurial subjective well-being. Third, based on the conservation of resources theory, this study brings entrepreneur mindfulness into the theoretical model of this study. However, mindfulness does not strengthen the access to resources, that is, it does not play a positive role in moderating the relationship between entrepreneurial identity and work-related problem-solving pondering. There may be other individual characteristic variables related to this research model. For example, an individual's pro-social motivation also affects the individual's job characteristics and changes in emotional resources (Kibler et al., 2019), which may enhance the work-related problem-solving pondering brought about by the entrepreneur's identity and bring role stress. Future studies should further examine the boundary conditions under which entrepreneurial identity affects the benefits or costs of entrepreneurs' subjective well-being. Finally, although this research explores the linear relationship between entrepreneurial identity and entrepreneurial subjective well-being based on resource conservation theory and is supported by empirical research, follow-up research can further investigate the non-linear relationship between the two, that is, the inverted U-shaped relationship. This is likely to be a research direction worthy of further advancement in future research.

## Data Availability Statement

The raw data supporting the conclusions of this article will be made available by the authors, without undue reservation.

## Ethics Statement

Ethical review and approval was not required for the study on human participants in accordance with the local legislation and institutional requirements. The patients/participants provided their written informed consent to participate in this study.

## Author Contributions

HY and LZ conceived and supervised the study. LZ wrote the manuscript. YW and HS improved the manuscript. All authors contributed equally to this manuscript and reviewed and approved this manuscript for publication.

## Funding

This research was funded by National Social Science Foundation of China (No. 19BSH110).

## Conflict of Interest

The authors declare that the research was conducted in the absence of any commercial or financial relationships that could be construed as a potential conflict of interest.

## Publisher's Note

All claims expressed in this article are solely those of the authors and do not necessarily represent those of their affiliated organizations, or those of the publisher, the editors and the reviewers. Any product that may be evaluated in this article, or claim that may be made by its manufacturer, is not guaranteed or endorsed by the publisher.
