# Supplementary material for: Benefits and Costs of Happy Entrepreneurs: The Dual Effect of Entrepreneurial Identity on Entrepreneurs' Subjective Well-Being
Source: Front Psychol. 2021 Oct 29;12:767164. doi: 10.3389/fpsyg.2021.767164 (PMC8589039; doi:10.3389/fpsyg.2021.767164)
Supplement: Supplementary file 1 [file Data_Sheet_1.docx]

Supplementary Material

# Appendix A: Measurement Concept and Code

Appendix A. Measurement Concept and Code

| Variables | Item # | Measure | Code |
| --- | --- | --- | --- |
| Subjective Well-being  (SWB) | SWB1 | In most ways my life is close to my ideal. | Using with a seven-point Likert scale, Variable’s value is calculated on the mean value from these items. |
|  | SWB2 | The conditions of my life are excellent. |  |
|  | SWB3 | I am satisfied with my life. |  |
|  | SWB4 | So far, I have gotten the important things I want in life. |  |
|  | SWB5 | If I could live my life over, I would change almost nothing. |  |
| Entrepreneurial Identity  （EI） | EI1 | In general, when someone praises entrepreneurs, it feels like a personal compliment. | Using with a seven-point Likert scale, Variable’s value is calculated on the mean value from these items. |
|  | EI2 | In general, when someone criticizes entrepreneurs, it feels like a personal insult. |  |
|  | EI3 | When I talk about entrepreneurs, I usually say ‘we’ rather than ‘they’. |  |
|  | EI4 | An entrepreneur’s successes are my successes. |  |
|  | EI5 | I am pleased to be a member of the entrepreneurial profession. |  |
|  | EI6 | If a story in the media criticizes entrepreneurs, I feel embarrassed. |  |
| Work-related Problem solving Pondering  （WPP） | WPP1 | After work I tend to think of how I can improve my work-related performance. | Using with a seven-point Likert scale, Variable’s value is calculated on the mean value from these items. |
|  | WPP2 | In my free time I find myself re-evaluating something I have done at work. |  |
|  | WPP3 | I think about tasks that need to be done at work the next day. |  |
|  | WPP4 | I find thinking about work during my free time helps me to be creative. |  |
|  | WPP5 | I find solutions to work-related problems in my free time. |  |
| Work-related Affective Rumination  （WAR） | WAR1 | I become tense when I think about work-related issues during my free time. | Using with a seven-point Likert scale, Variable’s value is calculated on the mean value from these items. |
|  | WAR2 | I am annoyed by thinking about work-related issues when not at work. |  |
|  | WAR3 | I am irritated by work issues when not at work. |  |
|  | WAR4 | I become fatigued by thinking about work-related issues during my free time. |  |
|  | WAR5 | I am troubled by work-related issues when not at work. |  |
| Mindfulness  (MIF) | MIF1 | I found it difficult to stay focused on what was happening in the present^*^. | Using with a seven-point Likert scale, Variable’s value is calculated on the mean value from these items. (*= reverse coded) |
|  | MIF2 | I rushed through activities without being really attentive to them^*^. |  |
|  | MIF3 | I did jobs or tasks automatically, without being aware of what I was doing^*^. |  |
|  | MIF4 | I found myself preoccupied with the future or the past^*^. |  |
|  | MIF5 | I found myself doing things without paying attention^*^. |  |
| Gender | Is the respondent male or female? | | “1”=Male,”0”=Female |
| Age | Age: <25 years、26 ~30 years、31 ~35years 、>36 years | | “1”= <25 years,  “2”=26~30years, “3”=31~35years,  “4”= >36 years. |
| Education | Education level：High school degree or below、Junior college degree、Bachelor degree、Master degree or above | | “1”=High school degree or below,  “2”=Junior college degree,  “3”=Bachelor degree，  “4”=Master degree or above. |
| Establishment | Start-up business years: ≤2 years、3~5years、>5years | | “1”= ≤2 years, “2”=3~5years,  “3”=>5years. |
| Number | Number of entrepreneurs：≤5、5~20、21~40、≥41 | | “1”=≤5,  “2”=5~20,  “3”=21~40,  “4”=≥41. |
| Industry | Industry：Entertainment industry、IT industry、Education industry、Retail industry、Other industry | | “1”=Entertainment industry,  “2”=IT industry,  “3”=Education industry,  “4”=Retail industry,  “5”=Other industry. |
